# Influence of Physical Modification of the Adhesive Composition on the Strength Properties of Aerospace Aluminum Alloy Sheet Adhesive Joints

**DOI:** 10.3390/ma15217799

**Published:** 2022-11-04

**Authors:** Izabela Miturska-Barańska, Anna Rudawska, Elżbieta Doluk

**Affiliations:** Department of Production Computerisation and Robotisation, Faculty of Mechanical Engineering, Lublin University of Technology, Nadbystrzycka 36, 20-618 Lublin, Poland

**Keywords:** mechanical properties of adhesives, adhesive testing, adhesive joints

## Abstract

One of the most important design factors in the constitution of adhesive joints is the correct choice of adhesive. Currently, there is a full range of options on the commercial market in this regard, but there is increasing research into modifying adhesives for specific engineering applications. The aim of this study was to analyze the effect of physical modification with fillers on the properties of the adhesive composition and the adhesive joints. The adhesives used in the study were a composition of Epidian 5 epoxy resin and PAC curing agent modified with 1% montmorillonite, 5% calcium carbonate and 20% activated carbon. The adhesive compositions in the cured state were subjected to strength tests and SEM and DSC analyses. Using these compositions, adhesive joints of EN AW 2024 T3 aluminum alloy sheets were also made. The tests carried out showed that, due to the use of different fillers, their effects on certain properties of the adhesive compositions are different types. It was shown that physical modification of the adhesive composition does not always result in positive effects. The study also attempted to determine the correlation between the properties of the adhesive compositions in the cured state and the strength of the adhesive joints.

## 1. Introduction

Assembly joints, i.e., made by welding, sealing, soldering, riveting or bonding, are used in almost every industry. One of the most recently developed methods of joining materials is bonding, which makes it possible to obtain structures that are much larger than could be made as a single component [1]. According to many authors described in their publications, such as Messler or Saboori [2,3,4], adhesive joints are also an alternative to other assembly joints in engineering applications because they have several advantages over conventional joining methods, while not altering the microstructure of the parts being bonded. The use of bonding technology allows for structures that are lighter in weight, yet have high strength, high fatigue life and reliability [5,6,7,8,9,10,11]. Structural bonding is, in many cases, one of the methods of relatively fast integration of machine parts, installations, vehicles or aircraft [9,10,12,13,14]. In addition to these mentioned fields, bonding technology is used in many industries such as construction, electrical engineering, medicine or light industries [15,16]. As Kinloch mentioned in his paper [17], with bonding technology being so widely used, adhesives are being increasingly challenged to develop new and beneficial performance properties. Key manufacturers of adhesives, as well as various research institutions, carry out continuous research work with the aim of obtaining adhesives with the most advantaged properties possible for a specific field [6,18,19,20,21,22]. Bonding technology is inherently an interdisciplinary field, requiring a fundamental understanding of mechanics, surface engineering and materials engineering, and the topics are still relevant and being intensively developed.

The strength properties of adhesive joints significantly depend on the technology used to make them, to a greater extent than in other joints used in engineering [8,23]. The large selection of adhesives produced by various manufacturers creates problems in selecting the right adhesive for the designed structure, especially as the properties of adhesives presented by manufacturers do not always characterize their most important features and are not always clear to the potential user. Researchers dealing with adhesion issues in the context of obtaining the highest possible properties for adhesive joints have at their disposal the main key variable characteristics, the modification of which leads to an increase in or control of the adhesion force between the adhesive and the component to be joined. Among these dependent variables are the chemical composition and properties of the adhesive composition and the surface stereometrics structure of the material to be bonded. The adhesive can be selected from commercially available compositions that exhibit the ability for Lifshitz–van der Waals intermolecular interactions, but also involving as little contribution as possible to permanent dipole interactions [24,25]. The adhesive can also be formulated as an acidic, basic or bifunctional bonding agent. Then, its properties, such as surface tension or viscosity, can be altered through the use of different types of additives, such as thixotropy agents [26]. Most adhesives used as structural adhesives are polymer compositions. For example, adhesives made from epoxy resins are designed so that internal crosslinking occurs leading to increased cohesive strength, but can also produce covalent bonds [27,28,29]. The durability of an adhesive joint depends mainly on the way it is loaded and the environment in which it is exposed [11,23,30]. Due to these aspects, numerous experimental, often destructive, experiments are conducted that address the strength of the adhesive compositions themselves, as well as adhesive joints made under varying structural and technological factors. The strength of an adhesive joint is one measure of the properties of adhesives [31,32]. An important direction of modern technology research is to subject adhesive compositions to modifications, particularly through the use of nanofillers, even a small addition of which can improve certain characteristics of adhesive materials [29,33,34,35,36]. Three types of modification in the literature are distinguished [11,37,38]: chemical, physical and physicochemical.

This paper presents the results of physical modification of epoxy adhesive compositions. Physical modification occurs through physical phenomena. Modified adhesives differ from those before modification in structure, physical properties, functional properties, visual properties, etc. The most common methods of physical modification are the addition of fillers. The performance properties of modified materials significantly depend on the type of filler used (particle shape and size, specific surface area, dispersed phase concentration) [39,40,41]. The best properties are obtained when the smallest possible fillers are introduced, preferably with particle sizes measured on the nanometer scale [42]. The authors of papers [43,44,45,46,47] have researched modifications related to bonding technology, but these mainly relate to surface modification issues and surface preparation of bonded components. Zheng, in one of his papers [48], states that the strength of the joint depends on the properties of the adhesive, but also on the adhesion between the adhesive and the binder. The effect of introduced fillers on the properties of adhesive compositions was presented in the work of Miturska et al. [34] where researchers described the results of a study on the effect of modification with natural fillers on the mechanical properties of epoxy adhesive compositions after storage time. In this study, the authors use two epoxy resins, Epidian 5 and Epidian 53, which were modified with a 2% addition of Montmorillonite, calcium carbonate and activated carbon, which were cured with the Mannich base. The compositions were cured for 7 days and seasoned for 4 months. Rudawska and Frigione in their work [49] present the effect of the aqueous environment on the mechanical properties of the epoxy resin Epidian 5 modified with calcium carbonate in amounts of 1%, 2% and 3% by weight of the resin. Since epoxy adhesives are one of the most common types of adhesives used in mechanical engineering, it seems reasonable to undertake research to determine the effect of physical modification of an epoxy adhesive composition on its mechanical and physical properties, as well as its effect on the strength properties of adhesive joints. In the various ways of modifying epoxy compositions, it should be taken into consideration that, while influencing the change in certain properties, others can be improved or degraded at the same time. As epoxy adhesives are one of the most commonly used structural adhesives, an attempt was made to modify them. The focus was on the use of modified adhesives in the context of bonding material that is used for aerospace structures. The aerospace industry is constantly developing and a lot of research is being carried out in this area, which makes this topic current and interesting from the point of view of both researchers and manufacturers. Aerospace manufacturers are looking for solutions to achieve lighter but stronger structures compared to conventional joints, such as riveted or welded joints. Structural bonding is currently widely used in the construction of aircraft airframes in the manufacture of components consisting of thin sheets and profiles, in the manufacture of sandwich structures, aircraft control components (rudders, ailerons), wing mechanization components, as well as in aircraft structures made of composites, where together with mechanical joints they form so-called hybrid joints. The large-scale introduction of structural adhesive joints has enabled aircraft weight reductions in the range of 10–15%. Examples of the use of adhesive joints in aviation include the Airbus A380 and Boeing 787 Dreamliner aircraft, as well as the bonding of the rotor blades of the Mi-2 helicopter’s carrier. This paper deals with the problem of epoxy adhesive compositions modification with fillers of various origin in the aspect of changing the mechanical properties of adhesive compositions, as well as some mechanical properties of aerospace aluminum alloy sheet adhesive joints. The adhesive compositions used in the study were made on the basis of the Epidian 5 epoxy resin. The choice of this resin is based on its properties, as it has excellent adhesion to most construction materials and, moreover, it is a basic resin, not pre-modified, which makes it an ideal base for physically modified adhesive compositions. The main purpose of using fillers in adhesive compositions is to improve certain performance properties. In addition to this, an important aspect, given the dynamic technological development, is also the environmental aspect, which is why three types of fillers were used in the study, both from the group of organic fillers: activated carbon (CWZ-22) and inorganic fillers—calcium carbonate (CaCO_3_) and montmorillonite (ZR2).

## 2. Materials and Methods

**Figure 1:**
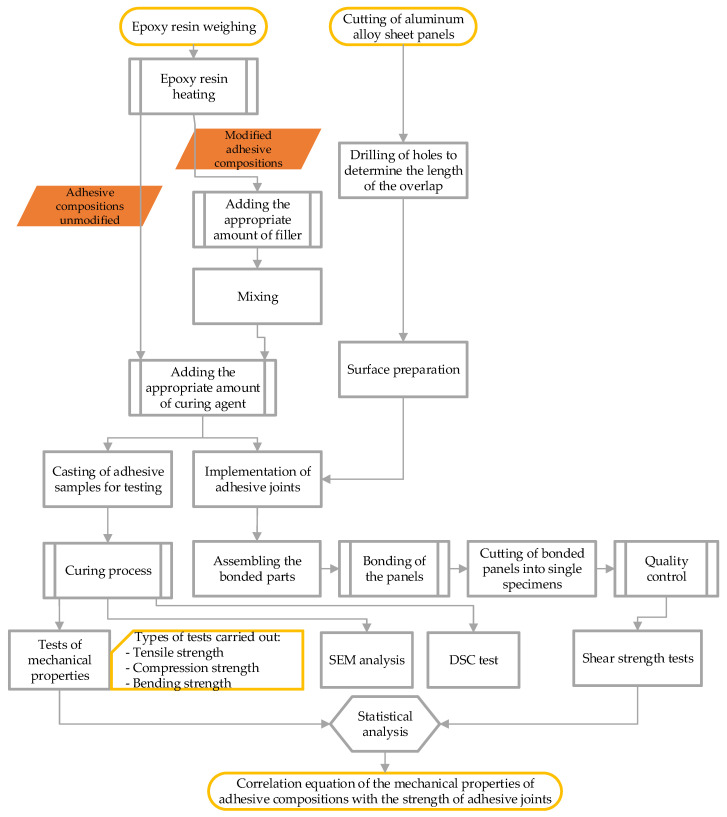
Flowchart of the sample making and testing procedure.

In this study, a number of tests were carried out on both adhesive compositions and adhesive joints made using these compositions. A flowchart of the sample making and testing procedure is shown in the Figure 1.

The detailed description and parameters of the various steps shown in the flowchart are described in the following subsections.

### 2.1. Adhesives

The adhesive used in the study was an epoxy adhesive based on bisphenol A epoxy resin with epichlorohydrin, cured with a polyamide curing agent and modified with three fillers: aluminosilicate modified with quaternary ammonium salt, calcium carbonate and particulate activated carbon.

Epoxy resin used in this study with the trade name Epidian 5 epoxy resin (CIECH S.A., Sarzyna, Poland) is a pure form of epoxy resin, which is a product of the reaction of bisphenol A with epichlorohydrin [38,50]. Adhesives prepared on the basis of this resin are used in metal bonding, in building structures as anti-corrosion and electro-insulating coatings. It is characterized by good dielectric and mechanical properties, minimal contraction during curing and high chemical resistance. The epoxy number of this resin is 0.48–0.52 mol/100 g, the viscosity measured at 25 °C is in the range 20,000–30,000 mPa∙s and the density measured at 20 °C is 1.16 g/cm^3^ [51,52].

The curing agent used in the study was a polyamide curing agent with the trade name PAC curing agent (CIECH S.A., Sarzyna, Poland) consisting of fatty acids, C18-unsaturated, dimers, polymeric reaction products with triethylenetetramine. This curing agent increases the elasticity and impact strength of the composition. It belongs to the group of slow-reacting curing agents and is, therefore, an excellent component for curing modified adhesive compositions. The amine number of this curing agent is 290–360 mg KOH/g, the viscosity measured at 25 °C is in the range 10,000–25,000 mPa∙s and the density measured at 20 °C is 1.10–1.20 g/cm^3^ [52,53]. The curing agent in the compositions used in this study was added at a stoichiometric ratio of 80 parts by weight per 100 parts by weight of resin.

Three types of fillers were used in the study. The choice of fillers was determined by the wide range of application possibilities. The first filler used was a filler with a high degree of fineness (i.e., a filler with a particle size on the micro and nano scale) with the trade name ZR2 NanoBent (Zakłady Górniczo-Metalowe “Zębiec” S.A., Zębiec, Poland). NanoBent ZR2 is an aluminosilicate modified with quaternary ammonium salt. It can be used as a dual-action additive: thixotropic and biocidal. The bulk density of ZR2 filler is less than 5 × 10^6^ g/m^3^. The ZR2 filler was 1 part by weight per 100 parts by weight of epoxy resin.

The second filler used in the study was calcium carbonate CaCO_3_ in powder form (Zakłady Przemysłu Wapienniczego Trzuskawica S.A., Siatkówka, Poland). The CaCO_3_ filler used in the study is free of any chemical structures associated with explosive properties, does not contain excess oxygen or any structural group tending to react exothermically with combustible material and is, therefore, classified as a non-explosive material without oxidizing properties. The bulk weight of CaCO_3_ is (0.7–1.4) × 10^6^ g/m^3^. The CaCO_3_ filler was 5 parts by weight per 100 parts by weight of epoxy resin.

The third filler used in the study was CWZ-22 activated carbon (PPH STANLAB SP. Z O.O., Lublin, Poland) in particulate form with a molar mass of 12.01 g/mol. Due to its properties, CWZ-22 is used as a catalyst and solid support for other catalysts as a component of gas scavengers and as a material to achieve large capacities in supercapacitors. The presence of carbon as a powder filler in a cured polymer matrix can significantly alter not only its thermal properties, but also its strength properties. Therefore, CWZ-22 is very widely used in many industries. The bulk weight of CWZ-22 is approximately 4 × 10^5^ g/m^3^. The CWZ-22 filler was 20 parts by weight per 100 parts by weight of epoxy resin.

For easier identification of the adhesives, in this paper the designations used are presented in Table 1.

The amounts of fillers used were selected on the basis of own experimental studies and a review of the literature [29,34,54,55,56,57,58,59,60,61]. The adhesive compositions were prepared immediately before use. In order to achieve a proper mixing of the components of the adhesive compositions used, the mixing stage was carried out in several steps:

1. The components of the mixtures were carefully weighed using a KERN CKE 3600-2 laboratory scale (Kern, Albstadt, Germany).
2. Heating of the epoxy resin using an electric heater—DEPILUX 400 (Activ, Wroclaw, Poland) to 50 °C in order to reduce its viscosity. The temperature of the heated resin was controlled using an electric thermometer (Amarell Electronic, Kreuzwertheim, Germany).
3. Addition of accurately weighed quantity of filler (for modified compositions).
4. Mechanical mixing using a Güde GTB 16/5 A mixer (Güde, Wolpertshausen, Germany) with a turbine dispersing disc mixer at 1170 rpm for 2 min with simultaneous venting to remove gas bubbles formed during mixing.
5. Addition of an accurately weighed quantity of curing agent.
6. Mechanical mixing carried out according to the technology described in step 4.

The mixing process was carried out under laboratory conditions at a temperature of 23 °C ± 2 °C and a relative humidity of 23% ± 3%. The adhesive compositions were used to prepare samples for testing the properties of the adhesive in the cured state and to prepare adhesive joints.

### 2.2. Adherend

The material used for the adhesive joints under study was the EN AW 2024 T3 aluminum alloy. This alloy has lower corrosion resistance and lower weldability compared to other aluminum alloys, but contains a high amount of copper and has very high strength—compared to, for example, AW 2014 alloy and high fatigue strength—which is why it is often used in aviation. The chemical composition of the alloy used is shown in Table 2. The mechanical properties of the used adherend are presented in Table 3.

- Yield strength—302.5 MPa;
- elongation—16.5%;
- hardness—123 HB;
- thermal conductivity—170 W/mK;
- thermal conductivity—2.78 g/cm^3^.

### 2.3. Adhesive Test Samples

All the samples used in the testing of the adhesive compositions’ properties were obtained in a casting process using specially prepared molds with shapes and dimensions that corresponded to the required geometry of the samples. The mechanical properties of the adhesive compositions were tested in tensile, compression and bending tests.

**Figure 2:**
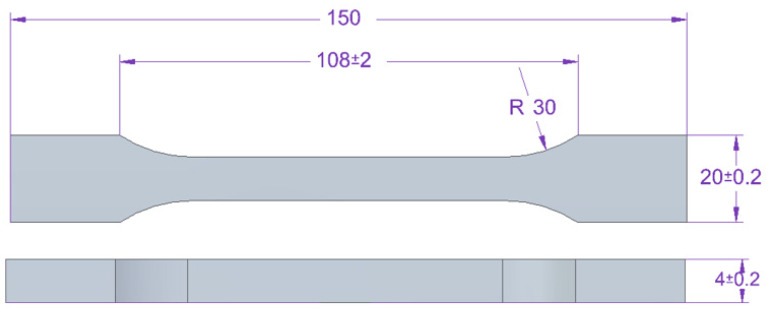
Shape and dimensions of the dump-bell type 1B sample of adhesive compositions for the tensile strength tests (all units are in millimeters).

For the tensile tests, dump-bell type 1B samples were used, in accordance with PN EN ISO 527-2 standard [63]. The dimensions of the samples used are shown in Figure 2.

Tensile testing of the adhesive compositions was carried out on a Zwick Roell Z150 testing machine (Zwick/Roell, Wroclaw, Poland), in accordance with PN EN ISO 527-1 standard [63]. The crosshead speed during the test was 5 mm/min. The initial tensile force was 30 N.

**Figure 3:**
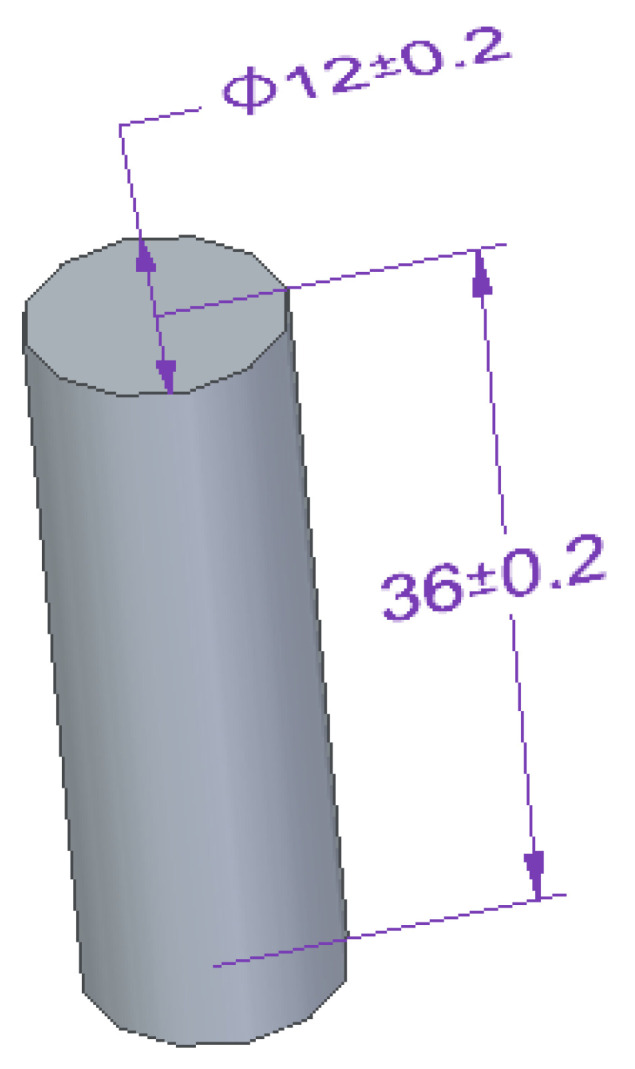
Shape and dimensions of the cylindrical sample of adhesive compositions for the compressive strength tests (all units are in millimeters).

Cylindrical samples were used for compressive strength testing, with a height-to-base ratio (3:1) [64]. Special care was taken during sample preparation to ensure that the bases of the samples were perpendicular to the direction of force application and parallel to each other. The dimensions of the cylindrical samples used are shown in Figure 3.

Compressive strength tests of the adhesive compositions were also carried out on a Zwick Roell Z150 testing machine (Zwick/Roell, Wroclaw, Poland). These tests were carried out in accordance with ISO 604 standard [65]. The assumed crosshead traverse speed during the test was 10 mm/min. The pre-test force was 20 N.

**Figure 4:**
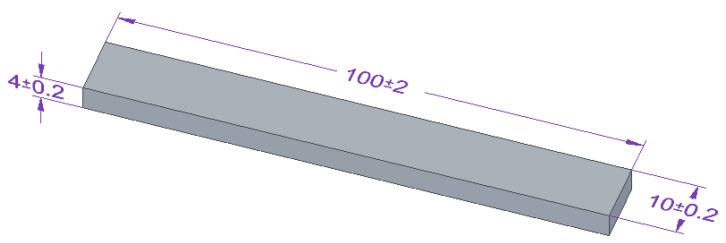
Shape and dimensions of the beam sample of adhesive compositions for the bending strength tests, in accordance with ISO 178:2003 standard [66] (all units are in millimeters).

For bending strength testing, beam-shaped samples were used. The dimensions of the bending strength test specimens are present in Figure 4.

According to the specifications [66], the dimensions used of 100 × 10 × 4 mm are suitable for the bending strength test in the three-point bending test, since the support spacing was 80 mm and, in addition, the recommended proportions for powder-filled enriched plastics: 10 mm ≤ b ≤ 25 mm and l ≥ 20 h were also followed.

Bending strength tests were carried out on a Zwick Roell Z2.5 testing machine (Zwick/Roell, Wroclaw, Poland), according to DIN-EN ISO 178 standard [66]. The test speed was 10 mm/min, and the initial test force was 5 N.

A series of 10 specimens was prepared for each adhesive composition.

In addition to the strength tests, the scanning electron microscopy (SEM) and differential scanning calorimetry (DSC) analyses were also carried out.

For SEM microscopic tests of the adhesive compositions, beam samples measuring 100 × 10 × 4 mm were used. The breakthroughs of the samples obtained by the percussion method were studied, which were then sputtered with gold using a Quorum Q150R ES—Spreading Deposition Rate (Quorum, Laughton, UK). The results of scanning electron microscopy (SEM) analysis were performed on the Tescan MIRA3 microscope (Tescan Orsay Holding, Brno-Kohoutovice, Czech Republic).

Samples of the adhesive compositions in the cured state for DSC differential scanning calorimetry analysis, weighing 6–12 mg, were taken from 100 × 10 × 4 mm beams. The analysis of modified epoxy composition using differential scanning calorimetry was carried out in accordance with EN ISO 11357-1 standard [67]. Special calibration files and samples made of light metals, such as indium, zinc, tin and bismud D > T, were used to calibrate the temperature scale. The tests were carried out in the air atmosphere in the temperature range from 20 °C to 220 °C, with a heating rate of 10 K/min and a cooling rate of 5 K/min. The tests were carried out using a DSC Phox 200 P instrument (NETZSCH, Selb, Germany). SEM tests were carried out at an accelerating voltage of 5.0 kV, an SE secondary electron detector was used to image the adhesive compositions and the samples were sputtered with gold for 10 min.

All samples were cured and seasoned under laboratory conditions identical to those of mixing for 7 days. The technological conditions for curing process have been selected on the basis of manufacturers’ guidelines and research literature [68,69].

### 2.4. Adhesive Joint Test Specimens

The adhesive joints used in the study were made in accordance with the requirements of ASTM D1002 standard [70]. The preparation plan for the adhesive joints according to the guidelines included several steps:

1. Cutting of aluminum sheets.

The components to be bonded were cut from EN AW 2024 T3 sheet metal with a thickness of 2.00 ± 0.12 mm to a dimension of 101.60 ± 0.25 mm × 177.80 ± 3.17 mm. Cutting was carried out in a hydroabrasive cutting process using a Waterjet Eckert Combo portal cutting machine (Eckert AS Sp. z o.o., Legnica, Poland). Cutting process speed—200 mm/min, water pressure—3500–105 Pa, nozzle distance from the material being cut—3 mm, abrasive flow rate during the cutting process—approx. 0.4 kg/min and abrasive material—Garnet sand of mesh 80 granulation.

2. Drilling of holes to determine the length of the overlap.

Two ϕ2.5-mm holes were drilled in each cut sheet for ground fixing pins, which enabled the panels to be assembled in a defined geometry in the next stage, while maintaining a constant overlap length of 12.7 ± 0.25 mm, as specified by the guidelines.

3. Surface preparation of the plates to be bonded.

The surface of the plates was prepared for bonding by sandblasting and degreasing with acetone. Sandblasting was carried out on a cabin sandblaster (Cormak, Siedlce, Poland) using Garnet abrasive granulation mesh 80 (Garnet Poland, Elbląg, Poland) with the following parameters:
  - Distance of the nozzle from the sample—h = 97 mm;
  - sandblasting speed—V = 53 mm/min;
  - pressure—P = 5–10^5^ Pa;
  - angle between specimen and direction of jet—90°;
  - number of specimen displacements—2.
Sandblasted parts were subjected to degreasing with technical acetone in a bath for 20 min and wiped with clean cleaning cloth, followed by a second washing and drying—self-drying in 10 min.

4. Assembling the panels.

After measuring the length of the overlap on the sheets to be bonded, the surface was secured with self-adhesive Teflon tape, which made it easier to remove excessive adhesive bleed. The adhesive was applied in a thin, homogeneous layer to the surfaces of the parts to be bonded using a spatula over the target length of the overlap.

5. Bonding of the panels.

The assembled components were bonded using the vacuum bag method with a constant pressure of 0.6—10^5^ Pa, which was realized using an SVAGG vacuum pump (Schunk, Lauffen/Neckar, Germany). The adhesive joints were cured for 7 days under laboratory conditions similar to the adhesive mixing.

6. Cutting of bonded panels into single specimens.

Cutting of the panels into specimens for testing the properties of the adhesive joints was carried out on a Waterjet Eckert Combo machine. The cut single-lap specimens were 25.4 ± 0.25 mm × 190.50 ± 0.25 mm.

7. Quality control.

**Figure 5:**
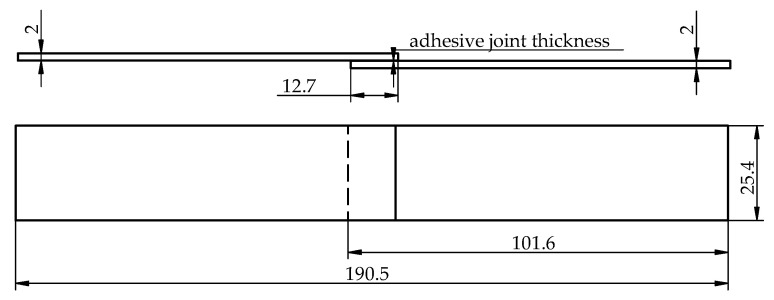
Geometry and dimensions of the single-lap adhesive joint used in the tests performed in accordance with ASTM D1002 standard (all units are in millimeters).

The cut-out single specimens of the adhesive joints were visually controlled to ensure that the bonding was correct and that excessive adhesive bleed was removed. The required dimensions were also checked. The required measurement accuracy and tolerances of the dimensions were obtained. In Figure 5, the dimensions of the tested adhesive joints are shown. The average thickness of the adhesive joint was 0.100 mm ± 0.025 mm.

The single-lap adhesive joints were subjected to strength tests on a Zwick Roell Z150 testing machine (Zwick/Roell, Wroclaw, Poland), in accordance with ASTM D1002 standard [70]. The crosshead speed during the test was 1.5 mm/min with an initial force of 5 N. The shear tensile strength was determined. Ten adhesive joint specimens were made for each adhesive composition.

## 3. Results

### 3.1. Strength Properties Test Results of Adhesive Compositions

To determine the effect of the filler on selected properties of the adhesive compositions, their properties were analyzed and compared with those of unmodified adhesives. The obtained test results are presented in Tables 4, 6 and 8. Statistical analysis of the obtained results was additionally carried out for a more accurate analysis and interpretation; the results of which are presented in Tables 5, 7 and 9. The statistical analysis presented in this paper was performed using Statistica 13.1 software.

Table 4 shows the results of the tensile strength test of the adhesive compositions.

Analyzing the tensile strength results of the tested adhesive compositions, it can be seen that the physical modification had a positive effect on the results obtained for two modified compositions. The highest average tensile strengths were obtained for E5/PAC/ZR2/100:80:1 adhesive composition—55.70 MPa. A lower strength of only 1.36% was obtained for E5/PAC/CaCO_3_/100:80:5 compositions—54.94 MPa. The reference (unmodified) E5/PAC/100:80 composition had an average strength of 53.86 MPa. The lowest strength was obtained for the E5/PAC/CWZ-22/100:80:20 composition—46.79 MPa—but in this case the repeatability of the results was at the highest level. The lowest repeatability of results was obtained with the reference E5/PAC/100:80 composition. However, in order to clearly assess the results obtained from the study, a more thorough statistical analysis was carried out. The normality of distribution and homogeneity of variance assumption was met, so a post hoc parametric test of significant differences was performed. The results of this test are shown in Table 5.

Based on the statistical analysis of the adhesive compositions’ tensile strength results, it can be seen that no statistically significant differences are found for compositions E5/PAC/100:80, E5/PAC/ZR2/100:80:1 and E5/PAC/CaCO_3_. At the assumed significance level α = 0.05, a statistically different average tensile strength was obtained for the E5/PAC/CWZ-22/100:80:20 composition. In order to clearly assess the results obtained from the study, a more thorough statistical analysis was carried out. The normality of distribution and homogeneity of variance assumption was met, so a post hoc parametric test of significant differences was performed. The results of this test are shown in Table 5.

Table 6 summarizes the compression strength test results.

Analyzing the compression test results, the positive effect of physical modification of the tested adhesive compositions can be seen. The highest average compression strength was obtained for the E5/PAC/CWZ-22/100:80:20—81.43 MPa. A lower average compression strength was obtained for the E5/PAC/CaCO_3_/100:80:5—77.04 MPa. A 1.13% lower average compression strength was obtained for the E5/PAC/ZR2/100:80:1—76.17 MPa. However, the results for samples of this composition had the highest repeatability, with a standard deviation of 0.98%. The lowest compression strength was obtained for the reference E5/PAC/100:80 composition—71.72 MPa—as well as the lowest repeatability of results—a standard deviation of 5.44%. The assumption of normality of distribution and homogeneity of variance was met, so the parametric Tukey statistical test was used to determine significant differences at the assumed significance level of α = 0.05. The results of this test are presented in Table 7.

Statistical analysis of the compression strength results of the adhesive compositions showed significant differences between the reference E5/PAC/100:80 composition and the others, and the E5/PAC/CWZ-22/100:80:20 composition and the others. No significant differences were observed for the E5/PAC/ZR2/100:80:1 composition and E5/PAC/CaCO_3_/100:80:5 composition. Thus, it can be concluded that modification with these two fillers causes a similar effect in terms of the compression strength of the compositions, while the greatest effect of the modification on the compression strength of the adhesive compositions was observed for the E5/PAC/CWZ-22/100:80:20 composition.

Table 8 shows the bending strength results obtained for the analyzed adhesive compositions.

Based on tests results, it can be seen that the highest bending strength was obtained for the E5/PAC/100:80 reference composition. E5/PAC/CaCO_3_/100:80:5 epoxy composition had a slightly lower bending strength by 3.2% of the average bending strength—79.29 MPa. The lowest failure strength in the three-point bending test was obtained for E5/PAC/ZR2/100:80:1 composition and E5/PAC/CWZ-22/100:80:20 composition. These compositions achieved strengths of 74.47 MPa and 74.35 MPa, respectively. In addition, the E5/PAC/CWZ-22/100:80:20 composition had the largest statistical dispersion of results, with a standard deviation of 4.76%. In order to further verify the differences between the results for each group, statistical analysis was carried out. The assumption that the distribution of test results conformed to a normal distribution was met. Likewise, the assumption of homogeneity of variance was verified with the appropriate statistical test—Levene’s test. Therefore, the Tukey post hoc test was used for further analysis.

The results of this test are presented in Table 9.

Statistical analysis of the bending strength test results showed that, at an assumed significance level of α = 0.05, there were no significant differences between the reference E5/PAC/100:80 composition and the E5/PAC/CaCO_3_/100:80:5 composition, and between the E5/PAC/ZR2/100:80:1 composition and E5/PAC/CWZ-22/100:80:20 composition.

### 3.2. SEM Analysis

From the research results of Fu et al. [42], it was concluded that the mechanical properties of particulate composites depend on the appropriate type of filler, on the interfacial interaction between matrix and filler, on the size of the particles used, on their distribution in the composite system and on their concentration of course.

**Figure 6:**
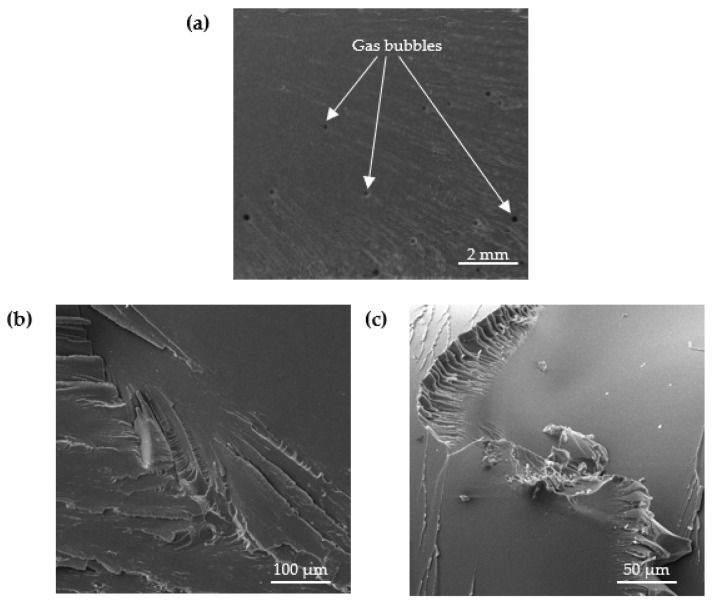
SEM images of fracture surface of E5/PAC/100:80 adhesive composition: (**a**) 9.78-mm view field, (**b**) 549-µm view field and (**c**) 241-µm view field.

**Figure 7:**
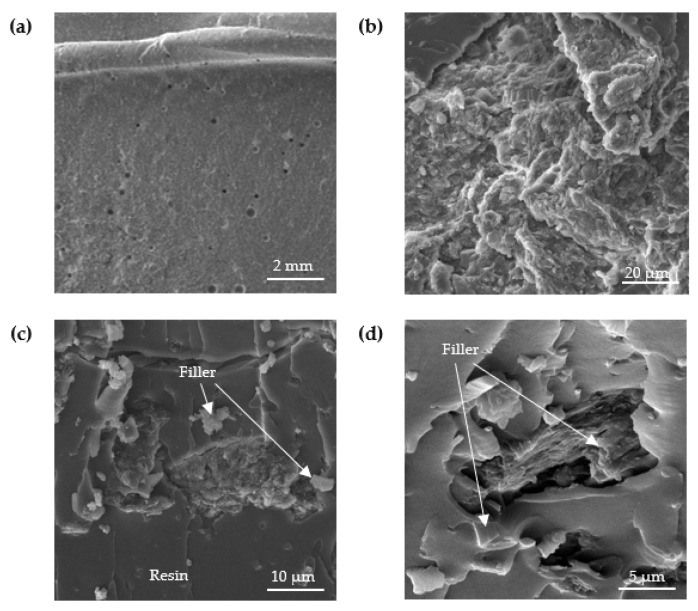
SEM images of fracture surface of E5/PAC/ZR2/100:80:1 adhesive composition: (**a**) 10.6-mm view field, (**b**) 69.1-µm view field, (**c**) 55.4-µm view field and (**d**) 23.1-µm view field.

**Figure 8:**
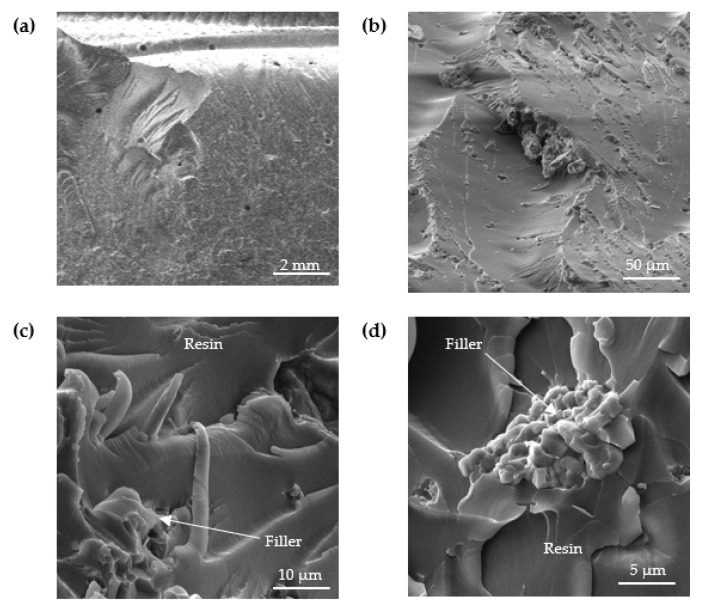
SEM images of fracture surface of E5/PAC/CaCO_3_/100:80:5 adhesive composition: (**a**) 10.5-mm view field, (**b**) 216-µm view field, (**c**) 43.9-µm view field and (**d**) 27.7-µm view field.

**Figure 9:**
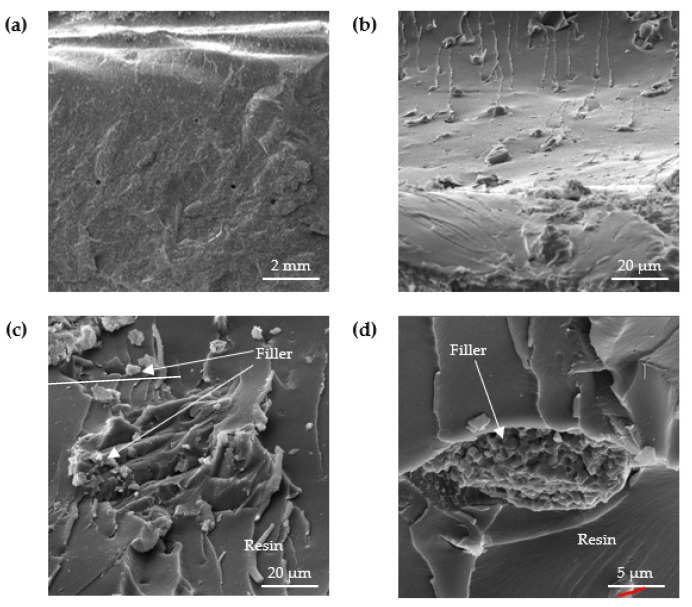
SEM images of fracture surface of E5/PAC/CWZ-22/100:80:20 adhesive composition: (**a**) 10.7-mm view field, (**b**) 153-µm view field, (**c**) 69.3-µm view field and (**d**) 27.7-µm view field.

The structure of the analyzed adhesive compositions in the cured state was studied by scanning electron microscopy (SEM). Figure 6, Figure 7, Figure 8 and Figure 9 show SEM images of the compositions studied.

SEM images in Figure 6 show that the unmodified E5/PAC/100:80 adhesive composition is characterized by a homogeneous, solid structure. Few gas bubbles are visible on the surface. In the case of the reference composition, the breakthrough is mild and malleable.

Analyzing the SEM images shown in Figure 7, a strong interaction of the filler with the matrix, i.e., the epoxy resin, can be seen. This shows that there is strong wettability at the interfacial surface of the filler and matrix in the structure of the composition. A large variation in the particle size of the filler can be observed, which consists of particles distinguished by an irregular, lamellar shape.

Analyzing the SEM images in Figure 8, it is possible to observe good wettability at the filler–matrix interface, which is due to the filler–matrix interaction seen in Figure 8d. An uneven distribution of the filler in the matrix can also be observed, which is a typical phenomenon for compositions subjected to physical modification with molecular fillers.

Figure 9 shows SEM images of E5/PAC/CWZ-22/100:80:20 composition. It can be seen that the impact test, after which the obtained breakthroughs were subjected to SEM analyses, resulted in the delamination of the dusty part of the carbon filler. In Figure 9c, a detailed image of the interaction between filler and matrix at the interfacial interface can be observed, from which it can be inferred that the filler is well wettable in the resin. It can also be seen from Figure 9d that there is delamination of the filler in the matrix.

### 3.3. Compositions Physical Properties Test Results

**Figure 10:**
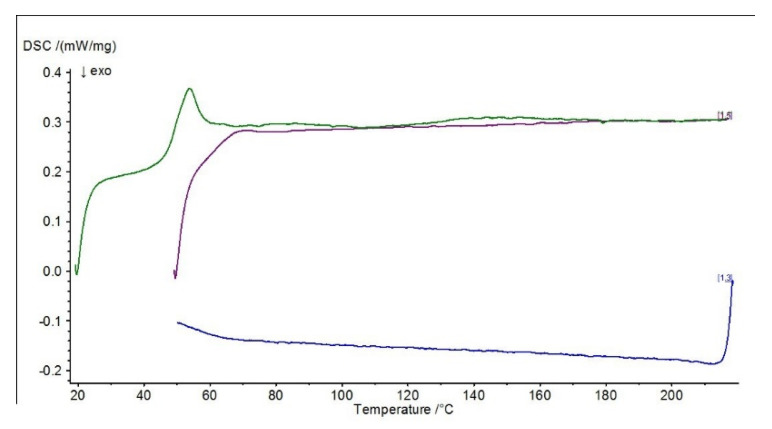
DSC diagram for E5/PAC/100:80 composition.

**Figure 11:**
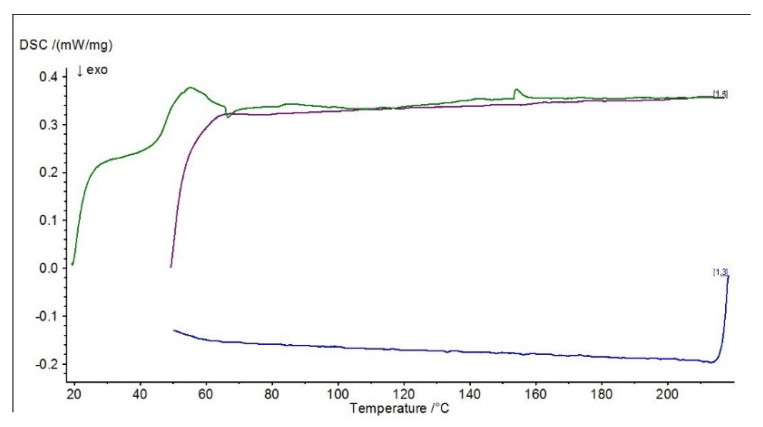
DSC diagram for E5/PAC/ZR2/100:80:1 composition.

**Figure 12:**
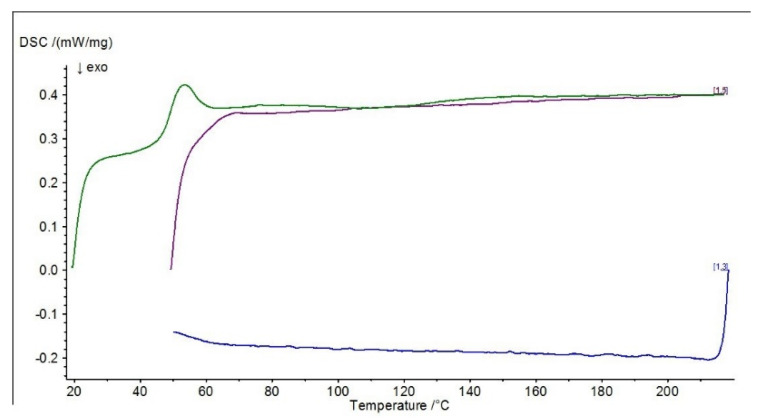
DSC diagram for E5/PAC/CaCO_3_/100:80:5 composition.

**Figure 13:**
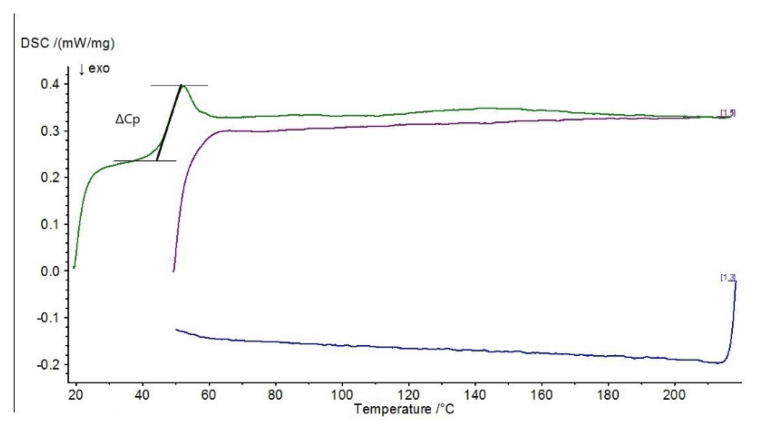
DSC diagram for E5/PAC/CWZ-22/100:80:20 composition.

Differential scanning calorimetry (DSC) was carried out to determine the effect of the modification on the physical properties of the adhesive composition, determined by varying the temperature (when the sample is heated and cooled at a specific rate). Differential scanning calorimetry diagrams are presented in Figure 10, Figure 11, Figure 12 and Figure 13.

The presented diagrams show the process of physicochemical changes in the test compositions under the influence of imposed temperature changes. The green curve in the DSC diagrams indicates the first heating range, the blue curve the cooling range and the purple curve the second heating range. Two heats of the system with the sample are carried out during the test. The first heating (indicated by the green line on the graphs) characterizes the melting effect, while the second heating (indicated by the purple line on the graphs) characterizes the glass transition effect. The test allows the glass transition temperature of the material to be determined, the method of which is shown graphically in Figure 13 (ΔCp).

In the DSC studies carried out, the characteristic temperatures of the modified adhesive compositions were determined. The glass transition temperature is one of the most important quantities characterizing the plastic properties of polymers. In the DSC curves presented, it is possible to distinguish sections of the so-called baseline, which are shifted parallel to the temperature axis. These mark the temperature intervals in which no heat release or absorption processes take place in the sample. When a reaction or phase transition occurs, the baseline changes to a peak—part of the curve deviates from the baseline and then returns to it. A distinction is made between an exothermic peak, when the temperature of the test sample is below the reference sample, and an endothermic peak, when the temperature of the test sample rises above the reference sample. In the case of an exothermic peak, heat must be supplied to the test sample (downwards peak), while in the case of an endothermic peak, the situation is reversed—heat is removed by the circuit, with the peak pointing upwards.

In the case of the reference E5/PAC/100:80 composition, a characteristic exothermic peak at the beginning of the DSC curves can be observed in both I heating and II heating. For the purple scale, II heating is shifted due to the fact that the temperature of the start of heating was higher, so the endothermic peak from I heating, which occurred at about 46 °C, is barely noticeable in II heating at 65 °C, indicating that the heat flux was quickly balanced. In addition, a second endothermic peak at around 120 °C can be observed in the first heating. For all adhesive compositions, characteristic endothermic peaks, i.e., related to heat release, can be observed. For the E5/PAC/ZR2/100:80:1 composition, an endothermic peak can be observed at 43–58 °C (which may indicate combustion or crystallization of filler molecules), followed by a small exothermic peak at 63 °C, and then a second peak can be observed, also endothermic at about 153 °C, which may indicate evaporation of the curing agent or other substance. Its formation may be related to the effect of the montmorillonite introduced into the resin. In the case of the E5/PAC/CaCO_3_/100:80:5 composition and E5/PAC/CWZ-22/100:80:20 composition, the first endothermic peak can also be observed at a temperature of about 43 °C and the second at about 120 °C.

The shape of the peak indicates the transformation taking place is also important. A sharp peak indicates that the transformation is taking place at a constant temperature; a fuzzy peak characterizes a transformation taking place over a certain temperature range.

### 3.4. Shear Strength of Single-Lap Adhesive Joints Test Results

**Figure 14:**
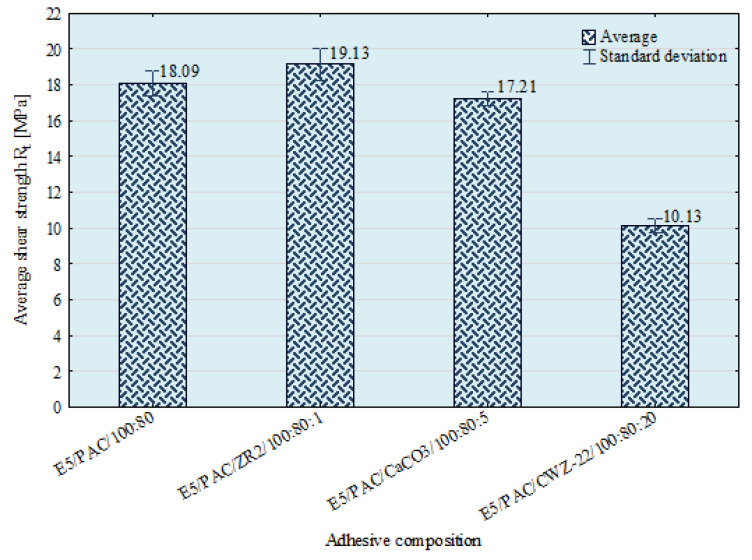
Shear strength (average values) of EN AW 2024 T3 aluminum alloy sheets single-lap adhesive joints.

The aim of this study was also to determine the effect of modifying the epoxy adhesive composition on selected mechanical properties of EN AW 2024 T3 aluminum alloy sheet adhesive joints. The results of the adhesive joints’ shear strength test are shown in Figure 14.

Analyzing the obtained results of the shear strengths of single-lap adhesive joints, it can be seen that the highest average strength values, above 19 MPa, were obtained for joints made with a E5/PAC/ZR2/100:80:1 composition. The lowest, almost twice lower, strengths were characterized by joints made with E5/PAC/CWZ-22/100:80:20 composition—10.13 MPa.

One of the problems considered in the study was whether the filler introduced into the adhesive composition increased the strength of the adhesive joints, so to be able to make an unambiguous assessment, the results obtained were subjected to more thorough statistical analysis. In the statistical analysis, a significance test was used to compare the average values of the test characteristics. As a first step, the Shapiro–Wilk W-test was used to check whether the distribution of the results in the separate groups follows a normal distribution. The results of this test are presented in Table 10.

Based on the results obtained, it can be seen that the condition of normality of distribution in all groups was not met (*p* < 0.05). Therefore, a non-parametric test for comparing multiple independent samples was used in further analysis. The Kruskal–Wallis test and the median test were applied. Assuming a significance level of α = 0.05, it was checked whether the average shear strength values of the adhesive joints for the different compositions did not differ significantly. The results of these tests are shown in Table 11 and Table 12.

For the Kruskal–Wallis test, the calculated significance level is less than the assumed α = 0.05, so it can be concluded that the obtained shear strength results differ significantly between groups. The median test can be interpreted similarly. Therefore, a test of multiple comparisons of average ranks for all trials was used to indicate where significant differences exist. The results of this test are shown in Table 13.

Based on the statistical analysis of the shear strength test results of adhesive joints, it can be concluded that significant differences exist between:

- E5/PAC/100:80 composition and E5/PAC/CWZ-22/100:80:20 composition;
- E5/PAC/ZR2/100:80:1 composition and E5/PAC/CaCO_3_/100:80:5 composition;
- E5/PAC/ZR2/100:80:1 composition and E5/PAC/CWZ-22/100:80:20 composition.
- No significant differences, however, were observed between:
- E5/PAC/100:80 composition and E5/PAC/ZR2/100:80:1 composition;
- E5/PAC/100:80 composition and E5/PAC/CaCO_3_/100:80:5 composition;
- E5/PAC/CaCO_3_/100:80:5 composition and E5/PAC/CWZ-22/100:80:20 composition.

Analysis of the results, therefore, shows that the addition of filler in the form of montmorillonite ZR2 and calcium carbonate CaCO_3_ in specific amounts in the adhesive composition used to make adhesive joints of EN AW 2024 T3 aluminum alloy sheets does not significantly alter the strength of the adhesive joints compared to the unmodified composition. The addition of activated carbon, on the other hand, significantly worsened the shear strength of the adhesive joints compared to the values determined for joints made with the reference composition. Considering the statistical analysis of the results of adhesive joints made with the modified compositions, it can be assumed that the most favorable results were obtained for adhesive joints made with E5/PAC/ZR2/100:80:1 composition.

## 4. Discussion

Based on the test results presented above and analyzing the results of the statistical analyses, it can be seen that the addition of CWZ-22 activated carbon in the composition in the amount of 20% adversely affects the tensile strength of the adhesive composition made based on Epidian 5 resin and PAC curing agent. For compositions with 1% montmorillonite ZR2 and 5% CaCO_3_ calcium carbonate filler, the modification resulted in an increase in tensile strength compared to the reference composition. The increase in the tensile strength values of the compositions and its change are very important, particularly for later structural joints, as the increase in the value of the longitudinal elastic coefficient is associated with an increase in the shear stresses in adhesive joints.

In compression strength tests, the addition of CWZ-22 activated carbon in the composition in the amount of 20% positively influences the compression strength of the adhesive composition made based on Epidian 5 resin and PAC curing agent. Similarly, in the case of compositions with 1% ZR2 montmorillonite and 5% CaCO_3_ calcium carbonate filler, the physical modification of the composition resulted in an increase in compression strength compared to the reference composition. It can also be seen that the introduction of the modifying additives improved the repeatability of the results, as the lowest repeatability of the results was obtained for the reference composition E5/PAC/100:80, as in the case of tensile strength.

The results of the bending strength determined by the three-point bending test and their statistical analysis show that the physical modification does not have a beneficial effect on the properties of the adhesive compositions. Only for the composition with 5% CaCO_3_ calcium carbonate filler the strength remained at a similar level. In addition, an additive of 20% activated carbon CWZ-22 in dust form reduced the repeatability of the test results. It can be concluded that the high filler content results in particle agglomeration, which, due to the lower contact surface area of the binder with the filler, contributes to a reduction in bonding capacity between filler and matrix. Therefore, this can affect significant changes in strength parameters in tests where the force is applied halfway along the sample length.

As the authors notice in their works [34,71], there is no known universal filler, the addition of which has only a positive effect on all the parameters of the adhesives evaluated according to the criterion adopted. In the opinion of Samal [72], the shape and size of the filler has a significant effect on the obtained properties of adhesive compositions. Kundie et al. [73] presents similar conclusions in their work, that the properties of modified compositions strongly depend on many factors, such as the type and mechanical properties of the fillers themselves, as well as the uniform dispersion of nanofillers in the polymer matrix. This can also be seen in the SEM microphotographs included in this paper.

For all modified adhesive compositions, there was a good interaction between the filler and the epoxy resin matrix, which is a basic assumption for the correct physical modification of the adhesive composition. The reason for this phenomenon can be seen in the relatively low viscosity, which was characterized by the matrix of the plastics, i.e., the epoxy resin Epidian 5. Lowering the viscosity of the resin was achieved by heating it to 50 °C during the preparation of the composition. According to Michels et al. [68], pre-exposure of epoxy to high temperatures accelerates curing and allows for a much faster development of strength and stiffness. This resulted in sufficient wetting of the filler in the epoxy resin, which facilitated the penetration of the resin particles between the filler particles. As also noted by authors such as Bittmann [74] in their paper, the wettability of filler surfaces plays an important role during physical modification. Matykiewicz [75] in her paper described the important role not only of the type of fillers, but also of the modification of the epoxy matrix to ensure good adhesion between all components in the composition to provide enhanced mechanical and thermomechanical properties of the hybrid composite.

The addition of modifying fillers reduces the amount of air bubbles in the structure of adhesive compositions in the cured state, which can affect the strength properties determined in static tests in which external forces are applied parallel to the specimen axis.

It can also be observed that the introduced fillers differ in particle shape: ZR2 montmorillonite has a lamellar structure, while CaCO_3_ calcium carbonate and CWZ-22 activated carbon particles are spherical. The fillers introduced into the composition tend to form agglomerates. The reasons for this phenomenon may lie in the interaction between the individual filler particles and in the filler–resin interaction and may affect the properties of the adhesive compositions.

In this paper, differential calorimetry studies were also carried out. As described by Moussa et al. [76] in their paper, DSC provides a method to effectively determine the glass transition temperature dependence of the degree of cure for structural adhesives. From the DSC plots presented, a shift in the glass transition temperature (purple line) towards a lower temperature (by about 5 °C) can also be observed for compositions modified with ZR-2 montmorillonite and CWZ-22 activated carbon compared to the reference composition. In the case of the composition modified with CaCO_3_ calcium carbonate, no changes were observed in the glass transition temperature region. In conclusion, it can be stated that the tested adhesive compositions are thermally stable, and in the case of the composition modified with 1% montmorillonite—E5/PAC/ZR2/100:80:1—there is a certain tendency for the curve to shift towards a higher temperature.

The results of the shear strength tests and their statistical analysis presented In this paper allow us to conclude that, for modified adhesive compositions intended for adhesive joints of aluminum alloy sheets, the type of filler used is important. In the case of the ZR2 NanoBent lamellar filler, a positive effect of the adhesive composition modification on the adhesive joint strength was observed. Considering the relatively low viscosity of the heated Epidian 5 resin, which is the matrix in the modified compositions, it can be concluded that adequate dispersion of the filler in the epoxy resin occurred, which consequently allows the penetration of resin particles between the filler particles. In the case of the composition modified with activated carbon, the strength of the joints deteriorated significantly compared to the other compositions. The reason for this may be too much modifier.

Due to the lack of a description in the literature to date of issues related to the correlation of the strength properties of the adhesive itself in relation to the strength of constituted adhesive joints, in the presented paper it was checked whether the strength of adhesive joints made with these compositions could be predicted from the properties of epoxy compositions in the cured state. An attempt was made to determine the correlation between the properties of the compositions in the cured state and the strength of adhesive joints. For this purpose, a linear multiple regression model was used. The purpose of multiple regression is to test the relationship between multiple independent variables and the dependent variable, so that it is possible to determine which independent variables have a significant effect on the dependent variable. Model verification in this case involves checking that the following model assumptions are met:

- the significance of the linear regression,
- significance of partial regression coefficients,
- no collinearity between independent variables,
- the assumption of constancy of variance, which means that the variance of the random component (residuals εt) is the same for all observations,
- no autocorrelation of the residuals,
- normality of the distribution of the residuals,
- the random component has an expected value equal to 0.

When there are strong correlations between the independent variables, the multiple regression function is statistically significant. This significance is verified by the F test, and for this test the probability level *p* should be less than the assumed significance level α.

The dependent variable in the case analyzed in this paper was the average shear strength of the adhesive joints (R_t_). The average strength test results of the adhesive compositions obtained in static tests, i.e., tensile strength (σ_m_), compression strength (σ_c_) and bending strength (σ_f_), were treated as the independent variables. It was assumed that there is a linear relationship between the variables, and the relationship has the formula:

(1) $$R_{t}=b_{0}+b_{1}\cdot \sigma_{m}+b_{2}\cdot \sigma_{c}+b_{3}\cdot \sigma_{f}\pm S_{e}$$

The task was to build a linear regression model, determining the coefficients of this equation—b_0_, b_1_, b_2_, b_3_—the standard error of the estimate S_e_. The number of estimated parameters was four, and the number of data was four. The results of the analyses are shown in Table 14.

A linear correlation coefficient R close to one was obtained, which indicates a linear relationship between the variables. The *p*-value for the F test was 0.018, which is less than the accepted significance test of α = 0.05, indicating the significance of the regression equation. The independent variables in Table 12 highlighted in red (i.e., compression strength and bending strength) are characterized by non-significant parameters *p* > 0.05, i.e., they are not correlated with the dependent variable. This may mean that these variables are collinear with another variable or are weakly correlated with the dependent variable. Therefore, these variables should be removed from the regression equation.

Analyzing the results obtained, it can be seen that the F value = 51.794, *p* < 0.1877, i.e., the regression equation is significant. The multivariate correlation coefficient is 0.99 and means that there is a strong linear relationship between the tensile strength of the adhesive compositions and the shear strength of the adhesive joints.

Based on the test performed, a multiple regression equation can be derived to determine the shear strength of the analyzed EN AW 2024 T3 aluminum alloy sheet adhesive joints from the tensile strength of the adhesive composition used to make the joints. However, it must be considered that this relationship applies only to compositions and joints prepared according to the technology described in this paper. The equation will be of the form:

(2) $$R_{t}=0.9807\cdot \sigma_{m}-35.65\pm 0.96$$

**Figure 15:**
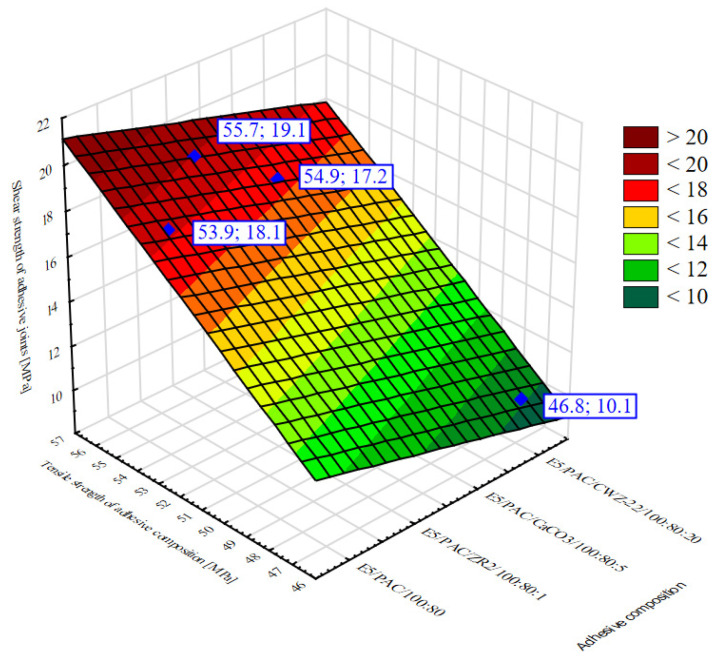
Area graph 3W of the dependence of the average shear strength of adhesive joints on the average tensile strength of adhesive compositions.

The data and the fitted surface are presented in Figure 15.

The graph presents the results of the tests, which were used to determine a multiple regression equation showing the dependence of the shear strength of the adhesive joints on the tensile strength of the adhesive compositions for the specific adhesive compositions. This makes it possible to predict the shear strength of the analyzed adhesive joints of aluminum alloy sheets, considering the tensile strength of the adhesive composition used to make the adhesive joint analyzed in this paper.

## 5. Conclusions

The experimental studies presented in this paper concerned the analysis of the mechanical and physical properties of selected modified adhesive compositions and the strength properties of EN AW 2024 T3 aluminum alloy sheet adhesive joints. Due to the use of different fillers, it was observed that their effects on specific properties of adhesive compositions are of different nature. It was shown that physical modification of the compositions does not always result in positive effects. Positive results of physical modification of adhesive compositions expressing an increase in strength parameters can be mentioned when the tensile strength and compression strength in the cured state are considered. In this case, the addition of fillers increased the strength parameters. When the impact and bending strength of the adhesive compositions were tested, the addition of the CWZ-22 activated carbon filler caused a deterioration in the results compared to the reference composition, and in addition, the results were also characterized by the lowest repeatability. It can be concluded that this is due to the high filler content, which can result in particle agglomeration. For the tensile and compression strength tests, the lowest repeatability was obtained for the unmodified composition. This may be due to the higher amount of air bubbles present in the plastic structure compared to the modified compositions, which affects the results of tests where external forces act axially to the test samples.

During the study, microscopic tests were also carried out on the structure of the compositions in the cured state. For all modified compositions, a good interaction between the filler and the epoxy resin matrix was evident, which is a basic assumption for the correct physical modification of an adhesive composition. It was observed that the best dispersion was obtained with the filler in the form of CWZ-22 activated carbon, as the filler in the matrix was delaminated in the structure of the plastic.

Thermal property tests conducted to determine the effect of temperature on the properties of the modified epoxy plastics showed that all adhesive compositions were thermally stable.

Tests on the strength properties of the adhesive joints showed that the addition of activated carbon had a significant effect on the deterioration of the shear strength of the adhesive joints compared to the results obtained for joints made with the reference composition. The analysis also showed that the addition of a filler in the form of ZR2 montmorillonite and CaCO_3_ calcium carbonate did not significantly alter the strength of adhesive joints of EN AW 2024 T3 aluminum alloy sheets compared to the unmodified composition. Composition with ZR2 montmorillonite caused the most favorable results.

The final stage of the experimental study was to verify whether the strength of adhesive joints made with these compositions could be inferred from the properties of the epoxy compositions in the cured state. The realized multiple regression model showed that there was a strong correlation between the tensile strength of the adhesive compositions and the shear strength of aluminum alloy sheet adhesive joints made with these compositions. The other strength properties of the adhesive compositions are not correlated with the adhesive joint strength. This correlation is described by relation (2).

By determining this correlation, it is possible to predict the strength of adhesive joints using knowledge of the strength properties of adhesive compositions, using the technology for making the joints as described in this paper, without the need to carry out destructive strength tests. However, it should be borne in mind that this relationship is specific to the materials and preparation technology of the adhesive compositions and adhesive joints used in the presented studies.

Epoxy adhesives are excellent structural adhesives with the advantage that they can be applied to dissimilar materials. Therefore, in future studies, the modified adhesive will be used for bonding other aluminum alloys as well as for bonding other materials.

Conclusions and findings from the studies show the need to intensify research work, especially in modifying epoxy adhesive compositions with fillers of organic and inorganic origin, in the aspect of using them in adhesive joints, and in the direction of determining the effect of different filler contents on the properties of modified epoxy adhesives. Future research will analyze the impact of modifying epoxy adhesives made with other epoxy resins and curing agents to produce more flexible adhesive joints. These adhesives will be used to bond structural materials other than aluminum alloy.

## Figures and Tables

**Table 1:** Designation of adhesive compositions used in the tests.

Epoxy Resin	Curing Agent	Filler	Amount of Filler (by Weight of Resin)	Designation of the Epoxy Composition
Epidian 5 (100 g)	PAC (80 g)	-	-	E5/PAC/100:80
		ZR2	1%	E5/PAC/ZR2/100:80:1
		CaCO_3_	5%	E5/PAC/CaCO_3_/100:80:5
		CWZ-22	20%	E57/PAC/CWZ-22/100:80:20

**Table 2:** Chemical composition of EN AW 2024 T3 aluminum alloy [62].

The Element	Si	Fe	Cu	Mn	Mg	Cr	Zn	Ti	Al
Contents, %	0.1671	0.2153	4.0975	0.4281	1.4405	0.0053	0.0154	0.0191	93.5699

**Table 3:** Mechanical properties of EN AW 2024 T3 aluminum alloy [62].

Mechanical Properties	Values
Tensile strength	447.2 MPa
Yield strength	302.5 MPa
Elongation	16.5%
Hardness	123 HB
Thermal conductivity	170 W/mK
Density	2.78 g/cm^3^

**Table 4:** Tensile strength of adhesive compositions.

Epoxy Adhesive Composition	Tensile Strength σ_m_ [MPa]				
	Average	Median	Quartile Range	Variation	Standard Deviation
E5/PAC/100:80	53.86	54.44	2.38	9.61	3.10
E5/PAC/ZR2/100:80:1	55.70	55.79	2.61	3.61	1.90
E5/PAC/CaCO_3_/100:80:5	54.91	54.88	1.92	4.47	2.11
E5/PAC/CWZ-22/100:80:20	46.79	46.35	0.85	0.97	0.99

**Table 5:** Significant difference in average tensile strength test results.

Epoxy Adhesive Composition		Tukey’s HSD Test for Average Values of Tensile Strength σ_m_ [MPa] at α = 0.05			
		{1} 53.86	{2} 55.70	{3} 54.91	{4} 46.79
E5/PAC/100:80	{1}		0.546	0.867	0.000
E5/PAC/ZR2/100:80:1	{2}	0.546		0.935	0.000
E5/PAC/CaCO_3_/100:80:5	{3}	0.867	0.935		0.000
E5/PAC/CWZ-22/100:80:20	{4}	0.000	0.000	0.000	

**Table 6:** Compression strength of adhesive compositions.

Epoxy Adhesive Composition	Compression Strength σ_c_ [MPa]				
	Average	Median	Quartile Range	Variation	Standard Deviation
E5/PAC/100:80	71.72	73.79	3.09	15.24	3.90
E5/PAC/ZR2/100:80:1	76.17	76.41	1.18	0.56	0.75
E5/PAC/CaCO_3_/100:80:5	77.04	77.41	1.28	0.90	0.95
E5/PAC/CWZ-22/100:80:20	81.43	80.88	2.28	1.67	1.29

**Table 7:** Significant difference in average compression strength test results.

Epoxy Adhesive Composition		Tukey’s HSD Test for Average Values of Compression Strength σc [MPa] at α = 0.05			
		{1} 71.72	{2} 76.17	{3} 77.04	{4} 81.43
E5/PAC/100:80	{1}		0.021	0.006	0.000
E5/PAC/ZR2/100:80:1	{2}	0.021		0.916	0.023
E5/PAC/CaCO_3_/100:80:5	{3}	0.006	0.916		0.006
E5/PAC/CWZ-22/100:80:20	{4}	0.000	0.006	0.023	

**Table 8:** Bending strength of adhesive compositions.

Epoxy Adhesive Composition	Bending Strength σ_f_ [MPa]				
	Average	Median	Quartile Range	Variation	Standard Deviation
E5/PAC/100:80	81.92	81.73	3.97	4.01	2.00
E5/PAC/ZR2/100:80:1	74.47	74.29	2.45	1.60	1.26
E5/PAC/CaCO_3_/100:80:5	79.29	79.64	1.10	0.70	0.84
E5/PAC/CWZ-22/100:80:20	74.35	75.34	4.14	12.52	3.54

**Table 9:** Significant difference in average bending strength test results.

Epoxy Adhesive Composition		Tukey’s HSD Test for Average Values of Bending Strength σ_f_ [MPa] at α = 0.05			
		{1} 81.92	{2} 74.47	{3} 79.29	{4} 74.35
E5/PAC/100:80	{1}		0.000	0.259	0.000
E5/PAC/ZR2/100:80:1	{2}	0.000		0.013	0.999
E5/PAC/CaCO_3_/100:80:5	{3}	0.259	0.013		0.011
E5/PAC/CWZ-22/100:80:20	{4}	0.000	0.999	0.011	

**Table 10:** Shapiro–Wilk W-test results for single-lap adhesive joints of EN AW 2024 T3 aluminum alloy sheets in groups.

Adhesive Composition	The Value of the W Statistic Shapiro–Wilk	The *p*-Value for the Shapiro–Wilk W-Test
E5/PAC/100:80	0.943	0.587
E5/PAC/ZR2/100:80:1	0.892	0.180
E5/PAC/CaCO_3_/100:80:5	0.813	0.021
E5/PAC/CWZ-22/100:80:20	0.859	0.074

**Table 11:** Results of non-parametric ANOVA rank Kruskal–Wallis test of the shear strength of single-lap adhesive joints of EN AW 2024 T3 aluminum alloy sheets divided according to the adhesive composition used.

Dependent: Average Shear Strength R_t_ [MPa]	Kruskal–Wallis Rank ANOVA; Average Shear Strength R_t_ [MPa] The Kruskal–Wallis Test: H (3, N = 40) = 32.34732 *p* = 0.0000		
	N Significant	Sum of Rank	Average Rank
E5/PAC/100:80	10	263.00	26.30
E5/PAC/ZR2/100:80:1	10	167.00	16.70
E5/PAC/CaCO_3_/100:80:5	10	55.00	5.50
E5/PAC/CWZ-22/100:80:20	10	335.00	33.50

**Table 12:** Test results of the median shear strength of single-lap adhesive joints of EN AW 2024 T3 aluminum alloy sheets, divided according to the adhesive composition used.

Dependent: Average Shear Strength R_t_ [MPa]	Median Test, Overall Median = 17.5759; Average Tensile Shear Strength R_t_ [MPa] Chi-Square = 23.20000 df = 3 *p* = 0.0000				
	E5/PAC/100:80	E5/PAC/ZR2/100:80:1	E5/PAC/CaCO_3_/100:80:5	E5/PAC/CWZ-22/100:80:20	Total
≤medians: observ.	3.00	0.00	7.00	10.00	20.00
expected	5.00	5.00	5.00	5.00	
obs.-exp.	−2.00	−5.00	2.00	5.00	
>medians: observ.	7.00	10.00	3.00	0.00	20.00
expected	5.00	5.00	5.00	5.00	
obs.-exp.	2.00	5.00	−2.00	−5.00	
Total: observed	10.00	10.00	10.00	10.00	40.00

**Table 13:** *p*-value for multiple comparisons test of average ranks for all shear strength tests of EN AW 2024 T3 aluminum alloy sheet adhesive joints.

Adhesive Composition	*p*-Value for Multiple Comparisons Kruskal–Wallis Test: H ( 3, N = 40) = 32.34732 *p* = 0.0000			
	E5/PAC/100:80	E5/PAC/ZR2/100:80:1	E5/PAC/CaCO_3_/100:80:5	E5/PAC/CWZ-22/100:80:20
E5/PAC/100:80		1.000	0.397	0.000
E5/PAC/ZR2/100:80:1	1.000		0.007	0.000
E5/PAC/CaCO_3_/100:80:5	0.397	0.007		0.193
E5/PAC/CWZ-22/100:80:20	0.000	0.000	0.193	

**Table 14:** Results of multiple regression.

N = 4	Summary of Dependent Variable Regression: Tensile Shear Strength R = 0.98123447 R^2^ = 0.96282108 Correct. R^2^ = 0.94423163 F(1,4) = 51.794 *p* < 0.01877 Estimation std. Error: 0.96454					
	b*	Err. std. of b*	b	Err. std. of b	t (2)	*p*
Free expression			−35.6	7.212	−4.942	0.038
Tensile strength of adhesive composition σ_m_ [MPa]	0.981	0.136	0.98	0.136	7.196	0.018
Compression strength of adhesive composition σ_c_ [MPa]	−0.807	0.417	−0.82	0.428	−1.935	0.192
Bending strength of adhesive composition σ_f_ [MPa]	0.446	0.632	0.488	0.691	0.705	0.553
Legend:						
b*	- standardized regression coefficients					
Err. std. of b*	- standard error of the coefficients b*					
b	- coefficients a_0_ and a_1_ of the regression equation ${\hat{y}}_{i}=a_{1}x+a_{0}+u_{i}$					
Err. std. of b	- standard error of calculated coefficients;					
t (2)	- quotient b/(Error std. of b);					
*p*	- computer significance level of the coefficients.					

## Data Availability

The raw/processed data required to reproduce these findings cannot be shared at this time due to technical or time limitations. Data can be made available on individual request.
